# Association of metalloestrogens exposure with depression in women across reproductive lifespan

**DOI:** 10.3389/fpsyt.2024.1486402

**Published:** 2024-12-03

**Authors:** Junjie Ren, Wanxin Wu, Jia Li, Qifang Hu, Mi Zhang, Jing Wang, Xiaoming Li, Yanwen Li, Binbin Huang

**Affiliations:** ^1^ Department of Medical Psychology, School of Mental Health and Psychological Science, Anhui Medical University, Hefei, Anhui, China; ^2^ Department of Maternal, Child and Adolescent Health, School of Public Health, Anhui Medical University, Hefei, Anhui, China; ^3^ Shenzhen Hospital of Southern Medical University, Shenzhen Clinical Medical School, Shenzhen, China; ^4^ Department of Materials Science and Engineering, Shanghai University of Engineering Science, Shanghai, China; ^5^ Department of Clinical Laboratory, Clinical Laboratory Shenzhen Longhua Maternity and Child Healthcare Hospital, Shenzhen, China

**Keywords:** metalloestrogens, depression, women, BKMR, NHANES

## Abstract

**Background:**

Exposure to metal could impact women’s depression risk. However, the connection and mechanisms between metalloestrogens exposure and depression are still not fully understood. We aim to explore the associations between metalloestrogens and the risk of depression in women across reproductive lifespan.

**Methods:**

Using data from NHANES 2011-2018, we employed logistic regression and baknernel machine regression (BKMR) to study links between metalloestrogen exposure and depression in US women. We analyzed how contraceptive use affects this relationship.

**Results:**

The study involved 3,374 adult women, with 345 of them experiencing depression. Our research revealed that certain metalloestrogens like Ba, Ca, Pb, Sb, and Sn were linked to higher depression risk in women, while Hg was associated with lower depression risk in older women. For women aged 18-44, a blend of metalloestrogens showed a significant positive correlation with depression risk, and the likelihood of depression in later years notably rose when the metal mixture concentration reached or exceeded the 60th percentile. Oral contraceptives would have an effect on the impact of metalloestrogen mixture exposure on depression in women during the reproductive stage.

**Conclusions:**

Our study indicates a significant link between metalloestrogen exposure and a higher risk of depression in adult women in the United States. This finding can aid in identifying the connection and enhancing women’s mental well-being.

## 1 Introduction

Depression, a prevalent mental illness, is marked by significant disability and mortality rates (1). Epidemiological studies indicate that major depression affects over 10 to 20 percent of the population, with more than 39 percent of individuals displaying suicidal tendencies (2). Moreover, depression stands as a key contributor to the global disease burden and is anticipated to become the second leading cause of disease burden by 2030 (3). Notably, gender variations in depression prevalence are substantial. For instance, research in China revealed a threefold higher prevalence of depression among women compared to men (4). In the U.S., depression rates among adolescents were approximately 25 percent for females and 10 percent for males (5). The reasons behind women’s susceptibility to depression remain unclear, yet studies suggest that women face a heightened risk of depression during hormonal fluctuations in specific reproductive stages like the perinatal and perimenopausal phases (6). This underscores the potential role of sex hormones in the biological predisposition to depression.

Estradiol (E2), also known as 17β-estradiol, is the most potent endogenous estrogen and a key sex hormone in the body (7). It is mainly produced by the ovaries during ovulation and plays a crucial role in regulating mood, cognitive function, and the immune system (8, 9). E2 has been shown to positively influence neurotransmitter systems such as glutamate, gamma-aminobutyric acid (GABA), 5-hydroxytryptophan (5-HT), and dopamine (10). Additionally, it controls and stimulates hypothalamus–pituitary–adrenals (HPA) axis activity, which is essential for stress response (11, 12). By modulating the emotional and cognitive systems involved in stress processing, E2 fluctuations may impact the risk of depression in women. Research indicates that women with depression tend to have lower E2 levels compared to healthy individuals (12). Animal studies have also demonstrated that abrupt E2 reduction post-ovariectomy can lead to increased depressive-like behavior in rats (13).

Metal ions have recently been discovered to imitate or interfere with natural estrogens, which interact with hormone receptors to produce agonistic or antagonistic endocrine effects (14). These inorganic xenoestrogens, known as metalloestrogens, include aluminum (Al), arsenic (As), barium (Ba), cadmium (Cd), chromium (Cr), cobalt (Co), copper (Cu), antimony (Sb), lead (Pb), mercury (Hg), molybdenum (Mo), nickel (Ni), selenium (Se), tin (Sn), and vanadium (V) (15). They are widespread in the environment and can infiltrate the body via food, water, or air (16). Organisms gradually accumulate metalloestrogens via the food chain or through the absorption of metallic elements. This bioaccumulation can lead to prolonged exposure, impacting the body’s endocrine system and causing hormonal disruption (17). Additionally, it may contribute to various health issues, including breast cancer, prostate cancer, endometriosis, reproductive disorders, and gestational diabetes (17, 18).

Recent studies have indicated a potential connection between metalloestrogens and a higher risk of depression in women. For instance, a cross-sectional study revealed a significant link between exposure to Cd and Pb and depression, particularly in women aged 20–59 (19). Another US study also showed a possible positive association between Cd levels in the blood and depression in adult women (20). Metalloestrogens can induce oxidative stress, disrupt neurotransmitter release, damage neurons, and harm the central nervous system (21). Furthermore, higher exposure to metalloestrogens may worsen inflammation, contributing to depressive episodes (22). These findings suggest that metalloestrogens might impact mood, cognitive function, and ultimately depression by influencing estrogen changes in the body.

However, current epidemiologic studies primarily focus on the effects of individual metalloestrogens on depression in women, neglecting the combined impact of multiple exposures. Women are not exposed to these substances in isolation; they encounter various environmental factors simultaneously. Metalloestrogens are commonly detected in urine and blood samples from the general population and may produce synergistic or cumulative effects (23). Thus, relying solely on individual exposure levels is inadequate for understanding depression’s development. Further research is necessary to examine the combined effects of metalloestrogens on depression risk. Additionally, since endogenous estrogen levels in women fluctuate with different reproductive stages and the use of external steroids like contraceptive use (24, 25), the impact of metalloestrogen exposure may also differ.

In this study, we analyzed data from the National Health and Nutrition Examination Survey (NHANES) to explore potential links between metalloestrogen exposure and depression in adult women in the United States across various reproductive stages. To evaluate interactions and determine overall effects, we applied several statistical methods, such as multivariate logistic regression and baknernel machine regression (BKMR). Our study is the first to explore how mixed exposure to various metalloestrogens affects depression in women, offering new insights into the relationship between metalloestrogen exposure and depression risk during different reproductive stages.

## 2 Study participants and methods

### 2.1 Study participants

The data utilized in this study were sourced from NHANES, a biennial survey carried out by the Centers for Disease Control and Prevention (CDC) to oversee public health in the nation. This database gathers a broad array of demographic, physiological, nutritional, and health-related information via periodic surveys of the health and nutrition status of the U.S. populace. Information is gathered through interviews conducted in homes and at mobile survey sites. Quality control measures, such as randomized repeat testing, are applied to laboratory samples to detect patterns, alterations, and uncertainties in the gathered data.

For our study, we procured data from four survey cycles spanning from 2011 to 2018, each lasting two years. We gathered information from women aged 18 years and older with full data on metalloestrogen exposure and details from the Depression and Reproductive Health Scale. Women with primary amenorrhea and pregnant individuals were excluded to precisely investigate the impact of metalloestrogens. In the end, 3,374 women were part of the analysis.

### 2.2 Methods

#### 2.2.1 Metalloestrogen exposure assessment

Urine samples were collected at the Mobile Examination Center (MEC) and then processed, stored, and transported to the Laboratory Sciences Division of the National Center for Environmental Health for analysis. We identified various metalloestrogens exhibiting relatively high estrogenic efficiency (15), such as Ba, Cd, Pb, Sb, Sn, Hg, and Mn (**Supplementary Table S1**), and quantified their concentrations in urine samples via Inductively Coupled Plasma Mass Spectrometry (ICP-MS). The laboratory techniques are detailed in the NHANES Official Instructions document. All test results reported adhere to environmental laboratory QC and QA standards, with comprehensive quality control and assurance guidelines outlined in the NHANES Laboratory/Medical Technician Procedures Manual (LPM).

The lower limits of detection (LOD) were 0.06 μg/L for Ba, 0.036 μg/dL for Ca, 0.13 μg/L for Mn, 0.03 μg/L for Pb, 0.022 μg/L for Sb, and 0.09 μg/L for Sn. The LOD for Hg was 0.13 ng/ml.

#### 2.2.2 Identification of depression

Depression was evaluated using the Patient Health Questionnaire-9 (PHQ-9), a reliable and valid tool in community samples for detecting the varying severity of depressive symptoms (26). The PHQ-9 comprises nine items assessing symptoms over the past two weeks. Each item is rated on a 4-point Likert scale from 0 to 3: 0 (not at all), 1 (a few days), 2 (no more than half the days), and 3 (almost every day). Total scores were calculated by summing all item scores. Participants with a total score of ≥10 were classified as having depressive symptoms (27).

#### 2.2.3 Covariates

The selection of covariates for this study was informed by previous research examining the association between exposure to environmental pollutants and depression (19). The covariates included age, defined according to the female reproductive lifespan, which begins with menarche and ends with menopause (24, 25, 28). Age categories were: 18-44 years as reproductive, 45-55 years as perimenopausal, and ≥56 years as the elderly group. Additional covariates were age at menarche, body mass index (BMI), diabetes mellitus, hypertension, and alcohol consumption (categorized as >12 drinks a year and ≤12 drinks a year).

### 2.3 Statistical analysis

When presenting basic data for women, continuous variables were shown as means and standard deviations, while categorical variables were shown as counts and percentages. The Pearson’s Chi-squared test and Wilcoxon rank sum test were utilized to compare differences in categorical and continuous variables between the depressed and non-depressed groups. As the metals had a skewed distribution, a log10 transformation was applied to normalize them. Spearman’s correlation analysis was used to examine the correlations between metals. Covariates included in all statistical models included age, drinking (number of drinks per year), diabetes, hypertension, age of menarche, family members, and BMI. In addition, we conducted subgroup analyses to examine potential age- and oral contraceptive-related variations in the associations between metalloestrogens exposure and depression.

Violin plots were employed to illustrate variations in the distribution of metal estrogens among age groups. Binary logistic regression modeling was utilized to investigate the correlation between metals and depression. Urinary levels of metalloestrogens were divided into quartiles (Q1-Q4), and the links between urinary metals and depression were analyzed across various concentration gradients. Statistical findings were presented as odds ratios (OR) and a 95 percent confidence interval (95% CI). Furthermore, a four-node restricted cubic spline was applied to observe the potential dose-response association between metals and depression.

In addition to examining the link between specific metals and depression, we applied BKMR to study how combined exposure to metalloestrogens relates to depression. We assessed the collective impact of metalloestrogen exposure on depression by maintaining all metalloestrogens within a set concentration range and observing outcomes with each 5% rise. We conducted 30,000 iterations for dependable estimates. Because major chronic disease (e.g., diabetes, hypertension) have effects on depression (29, 30), we conducted another separate sensitivity analysis by excluding participants with these diseases.

Statistical analysis was performed using SPSS 25.0 and R (version 4.2.3), with the utilization of the “ggcorrplot”, “gtsummary” and “bkmr” packages for the respective analyses. The significance level chosen for the study was P < 0.05.

## 3 Results

### 3.1 Population characteristics and metalloestrogens distribution

Throughout 2011 to 2018, NHANES included 19851 women participants. Initially, we excluded individuals under 18 years old (n = 6366), those with unreliable or missing health questionnaire and laboratory data (n = 9595), pregnant women (n = 233), primary amenorrhea (n = 4), and individuals with uncertain or missing values for other variables (n = 279). Ultimately, our sample comprised 3374 American women, among whom 345 were identified as experiencing depression (**Supplementary Figure S1**). The women’s baseline characteristics are presented in **Table 1**. 44% were aged 18-44 years, 18% were aged 44-55 years, and 38% were 56 years of age or older. Among depressed patients, 40% were in the 18-44 age group (reproductive years), 22% were in the 45-55 age group (perimenopausal years), and 38% were 56 years and older (old age). Age at menarche, BMI, hypertension, diabetes, and oral contraceptives varied between depressed and non-depressed groups (p<0.05).

**Table 1:** Basic characteristics of participants by depression in the U.S. women, NHANES 2011–2018.

Characteristic	Overall, N = 3374	No Depression, N = 3029	Depression, N = 345	*p*-value
Drinking in a year, n (%)				0.081
≤12 times	1971 (58%)	1750 (58%)	221 (64%)	
>12 times	1279 (38%)	1166 (38%)	113 (33%)	
Missing	124 (3.7%)	113 (3.7%)	11 (3.2%)	
Age of Menarche, Mean (SD)	12.69 (1.82)	12.71(1.80)	12.49 (1.95)	0.016
BMI, n (%)				<0.001
<18.5 kg/m^2^	75 (2.2%)	68 (2.2%)	7 (2.0%)	
18.5-24.9 kg/m^2^	1093 (32%)	1,019 (34%)	74 (21%)	
25.0-29.9 kg/m^2^	1034 (31%)	937 (31%)	97 (28%)	
≥30 kg/m^2^	1172 (35%)	1005 (33%)	167(48%)	
Hypertension, n (%)				<0.001
No	2197 (65%)	2,022 (67%)	175(51%)	
Yes	1177 (35%)	1,007 (33%)	170(49%)	
Diabetes, n (%)				<0.001
No	2875 (85%)	2615 (86%)	260 (75%)	
Yes	403 (12%)	327 (11%)	76 (22%)	
Missing	96 (2.8%)	87 (2.9%)	9 (2.6%)	
Age, n (%)				0.064
18-44 years	1,488 (44%)	1351 (45%)	137 (40%)	
44-55 years	610 (18%)	533 (18%)	77 (22%)	
≥56 years	1276 (38%)	1145 (38%)	131 (38%)	
Oral Contraceptive, n (%)				0.009
No	153 (34%)	1057 (35%)	96 (28%)	
Yes	2221 (66%)	1972 (65%)	249 (72%)	
Family members, Mean (SD)	3.08 (1.73)	3.09 (1.73)	3.00 (1.78)	0.13

N, number; SD, Standard Deviation.

**Figure 1:**
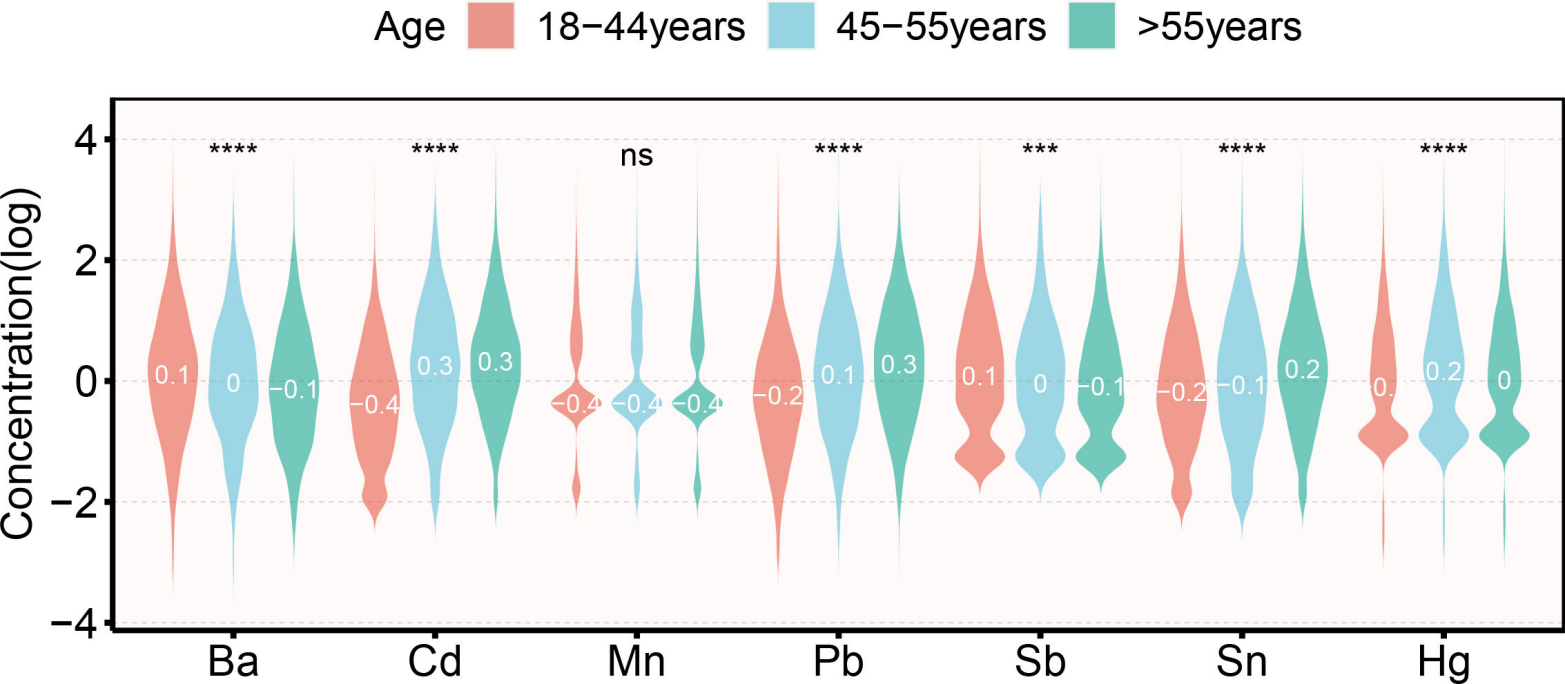
Violin plot of the distribution of metalloestrogens in different women reproductive cycles. ***: p < 0.001; ****: p < 0.0001; ns: p > 0.05.

The correlation heatmaps for the seven metallestrogens are displayed in **Supplementary Figure S2**. Strong positive correlations were observed between Sb, Pb, and all other metals. Correlations across all metals varied from 0.23 to 0.6. **Figure 1** illustrates the distribution characteristics of Log10 transformed metalloestrogens across various age groups. Ba, Cd, Pb, Sb, Sn, and Hg exhibit significant differences among the three age groups.

### 3.2 Association between individual metalloestrogen exposure and depression

Using logistic regression to explore the association between metalloestrogens and depression (**Table 2**). Ba and Sb showed a positive association with the risk of depression in the crude model (Ba, Crude OR 95%CI: 1.04 (1~1.08) *; Sb, Crude OR 95%CI: 2.78 (1.29~6) **). Cd and Pb showed positive associations with the risk of depression in both crude and corrected models (Cd, Crude OR 95%CI: 1.59 (1.32~1.93) ***, OR 95%CI: 1.62 (1.32~1.93) ***; Pb, Crude OR 95%CI: 1.4 (1.21~1.63) ***, OR 95%CI: 1.51 (1.29~1.77) ***). In addition, the second, third, and fourth quartiles of metalloestrogens were associated with a higher risk of depression compared to the lowest quartile (Cd, Q3, OR 95% CI: 1.51 (1.06~2.13) *, Q4 OR 95%CI: 1.98 (1.4~2.8) ***; Pb, Q3 OR 95%CI: 1.75 (1.22~2.5) **, Q4 OR 95%CI: 2.28 (1.61~3.23) ***; Sb, Q4 OR 95%CI: 1.84 (1.33~2.55) ***; Sn, Q2 OR 95%CI: 1.63 (1.11~2.4) *, Q3 OR 95%CI: 1.79 (1.22~2.62) **, Q4 OR 95%CI: 2.71 (1.88~3.91) ***.

**Table 2:** Association of metalloestrogens with depression, NHANES, 2011–2018.

Variables	Crude OR (95%CI)	OR (95%CI)^1^	OR (95%CI)^1^				P for trend
ug/L	Continuous		Q1	Q2	Q3	Q4	
Ba	1.04 (1~1.08)*	1.03(1~1.07)	Reference	1.36 (0.98~1.89)	1.24 (0.89~1.74)	1.36 (0.98~1.9)	0.129
Cd	1.59 (1.32~1.93) ***	1.62(1.32~1.93) ***	Reference	1.02 (0.71~1.47)	1.51 (1.06~2.13) *	1.98 (1.4~2.8) ***	<0.001
Mn	1.12 (0.99~1.28)	1.09(0.96~1.25)	Reference	0.9 (0.6~1.35)	1.2 (0.79~1.81)	/	0.071
Pb	1.4 (1.21~1.63) ***	1.51(1.29~1.77) ***	Reference	1.33 (0.92~1.92)	1.75 (1.22~2.5) **	2.28 (1.61~3.23) ***	<0.001
Sb	2.78 (1.29~6) **	2.25 (0.99~5.15)	Reference	1.05 (0.74~1.5)	1.06 (0.75~1.51)	1.84 (1.33~2.55) ***	<0.001
Sn	1.02 (0.99~1.04)	1.01 (0.98~1.03)	Reference	1.63 (1.11~2.4) *	1.79 (1.22~2.62) **	2.71 (1.88~3.91) ***	<0.001
Hg ^2^	0.87 (0.73~1.04)	0.9 (0.76~1.06)	Reference	0.67 (0.34~1.36)	0.83 (0.41~1.7)	0.68 (0.33~1.4)	0.947

Since the 25th percentile of Mn is equal to the 50th percentile, Mn is divided into three categorical variables.

^1^Adjusted for age, diabetes mellitus, hypertension, household size, age at menarche, number of drinks in a year, and BMI. Q, quartile.

^2^ ng/ml.

*: p < 0.05; **: p < 0.01; ***: p < 0.001.

Bold indicates p < 0.05.

We also stratified by age, exploring the relationship between metalloestrogen exposure and depression during the reproductive years (18-44 years), perimenopause (45-55 years), and old age (>55 years) (**Table 3**). Logistic regression found that Pb was positively associated with the risk of depression in reproductive women (Crude OR 95%CI: 1.34 (1.08~1.66) **, OR 95%CI: 1.4 (1.11~1.77) **). Ba, Cd, Pb, and Sb were positively associated with the risk of depression in perimenopausal women (Ba, crude OR 95%CI: 1.11 (1~1.22) *; Cd, Crude OR 95%CI: 1.99 (1.35~2.93) **, OR 95%CI: 2.19 (1.43~3.36) ***; Pb, Crude OR95%CI: 1.66 (1.03~2.69) *, OR 95%CI: 2.1 (1.21~3.66) **; Sb, Crude OR 95%CI: 9.5 (1.56~57.7) *, OR 95%CI: 12.1 (1.64~89.44)*). Cd and Pb were positively correlated with the risk of depression in old age (Cd, Crude OR 95%CI: 1.43 (1.11~1.83) **, OR 95%CI: 1.49 (1.15~1.93) **; Pb, Crude OR 95%CI: 1.39 (1.07~1.81) *, OR 95%CI: 1.57 (1.2~2.06) **. The highest tertile of Cd in any period was associated with a higher risk of depression compared to the lowest tertile (Q4: 18-44years OR 95%CI: 1.72 (1.01~2.95) *; 45-55years OR 95% CI: 1.39 (1.09~1.76) **; >55years OR 95%CI: 2.1 (1.24~3.55) **). Higher quartiles of Pb in any period were associated with a higher risk of depression compared to the lowest quartile (18–44 years, Q3 OR 95%CI: 2.59 (1.44~4.64). **, Q4, OR 95%CI: 2.41 (1.33~4.36) **; 45-55 years, Q4 OR 95%CI: 2.3 (1.09~4.82) *; >55 years, Q3 OR 95%CI: 1.73 (1~2.98) *, Q4, OR 95%CI: 1.94 (1.12~3.35) *). Compared with the lowest quartile, the highest quartile of Sb in the reproductive period was positively associated with the risk of depression (OR 95%CI: 2.45 (1.45~4.12) **). Compared to the lowest quartile, the high quartile of Sn in any period was positively associated with the risk of depression (18–44 years, Q2 OR 95%CI: 2.55(1.37~4.73) **, Q3 OR 95%CI: 2.45(1.31~4.58) **, Q4 OR 95%CI: 2.37(1.27~4.42) **. 45-55years, Q4, OR 95%CI: 4.43(1.88~10.44) **; >55years, Q3, OR 95%CI: 2.09 (1.16~3.76) *, Q4 OR 95%CI: 2.69 (1.51~4.79) **. Compared with the lowest quartile, the higher quartile of Hg in old age was negatively associated with the risk of depression. (Q2, OR 95%CI: 0.37 (0.14~0.97) *; Q4, OR 95%CI: 0.32 (0.11~0.88) *). In all women, the dose-response relationship between metalloestrogens and depression is shown in **Supplementary Figure S3**. All seven metalloestrogens showed linear associations with depression (p-non-linear > 0.05). In the sensitivity analyses, exclusion of the women with diabetes and hypertension (**Supplementary Tables S2– S7**) found similar results.

**Table 3:** Association of metalloestrogens with depression after age subgroup, NHANES, 2011–2018.

Variables	Crude OR (95%CI)	OR (95%CI)^1^	OR (95%CI)^1^				P for trend
ug/L	Continuous		Q1	Q2	Q3	Q4	
Ba							
18-44	1.04 (0.99~1.09)	1.03(0.98~1.08)	Reference	1.2(0.72~2.02)	1.18(0.7~2)	1.09 (0.64~1.84)	0.806
45-55	1.11 (1~1.22) *	1.1 (0.99~1.22)	Reference	2.03 (0.95~4.36)	1.62 (0.75~3.51)	1.61 (0.74~3.48)	0.397
≥56	1.02 (0.94~1.1)	1.03 (0.96~1.1)	Reference	1.1 (0.64~1.9)	1.11 (0.64~1.92)	1.6 (0.95~2.71)	0.088
Cd							
18-44	1.56 (0.94~2.59)	1.62(0.91~2.86)	Reference	1.09(0.62~1.88)	1.34(0.78~2.3)	1.72(1.01~2.95) *	0.032
45-55	1.99 (1.35~2.93) **	2.19(1.43~3.36) ***	Reference	1.52 (0.67~3.45)	1.65 (0.73~3.72)	1.39(1.09~1.76) **	0.008
≥56	1.43 (1.11~1.83) **	1.49(1.15~1.93) **	Reference	1.02 (0.57~1.83)	1.28 (0.74~2.23)	2.1 (1.24~3.55) **	0.003
Mn							
18-44	1.07 (0.75~1.52)	1.03 (0.71~1.5)	Reference	1.02(0.54~1.93)	1.25(0.65~2.39)	/	0.331
45-55	1.03 (0.77~1.38)	1.03 (0.71~1.5)	Reference	0.56 (0.23~1.33)	0.97 (0.4~2.3)	/	0.336
≥56	1.19 (0.98~1.44)	1.17 (0.93~1.46)	Reference	1.08 (0.53~2.2)	1.37 (0.67~2.83)	/	0.214
Pb							
18-44	1.34 (1.08~1.66) **	1.4(1.11~1.77) **	Reference	1.88(1.02~3.45)	2.59(1.44~4.64) **	2.41(1.33~4.36) **	0.002
45-55	1.66 (1.03~2.69) *	2.1 (1.21~3.66) **	Reference	0.94 (0.41~2.16)	1.7 (0.81~3.57)	2.3 (1.09~4.82) *	0.009
≥56	1.39 (1.07~1.81) *	1.57 (1.2~2.06) **	Reference	1.22 (0.69~2.17)	1.73 (1~2.98)*	1.94 (1.12~3.35) *	0.008
Sb							
18-44	2.19 (0.84~5.72)	1.45 (0.51~4.11)	Reference	1.18 (0.66~2.1)	0.95 (0.52~1.72)	2.45(1.45~4.12) **	0.001
45-55	9.5 (1.56~57.7) *	12.1(1.64~89.44) *	Reference	0.77 (0.34~1.74)	1.41 (0.67~2.95)	1.81 (0.89~3.68)	0.037
≥56	2.54 (0.43~15.01)	2.41 (0.32~18.38)	Reference	1.27 (0.72~2.24)	1.05 (0.59~1.87)	1.64 (0.96~2.82)	0.111
Sn							
18-44	1.01 (0.97~1.05)	1 (0.95~1.05)	Reference	2.55(1.37~4.73) **	2.45(1.31~4.58) **	2.37(1.27~4.42) **	0.027
45-55	1.02 (0.98~1.07)	1.03 (0.98~1.08)	Reference	1.53 (0.61~3.81)	1.85 (0.75~4.58)	4.43(1.88~10.44) **	<0.001
≥56	1.01 (0.98~1.05)	1.01 (0.97~1.04)	Reference	1.26 (0.67~2.37)	2.09 (1.16~3.76) *	2.69 (1.51~4.79) **	<0.001
Hg ^2^							
18-44	0.91 (0.69~1.19)	0.92 (0.7~1.2)	Reference	1.3 (0.38~4.48)	1.21 (0.34~4.27)	1.32 (0.38~4.63)	0.89
45-55	0.87 (0.64~1.19)	0.89 (0.65~1.22)	Reference	0.88 (0.1~7.65)	0.89 (0.1~7.92)	0.85 (0.09~7.67)	0.913
≥56	0.78 (0.54~1.11)	0.82 (0.58~1.15)	Reference	0.37 (0.14~0.97) *	0.46 (0.17~1.25)	0.32 (0.11~0.88) *	0.354

Since the 25th percentile of Mn is equal to the 50th percentile, Mn is divided into three categorical variables.

^1^Adjusted for, diabetes mellitus, hypertension, household size, age at menarche, number of drinks in a year, and BMI. Q, quartile.

^2^ng/ml

*: p < 0.05; **: p < 0.01.

Bold indicates p < 0.05.

### 3.3 Association of metalloestrogens mixture exposure with depression

**Figure 2:**
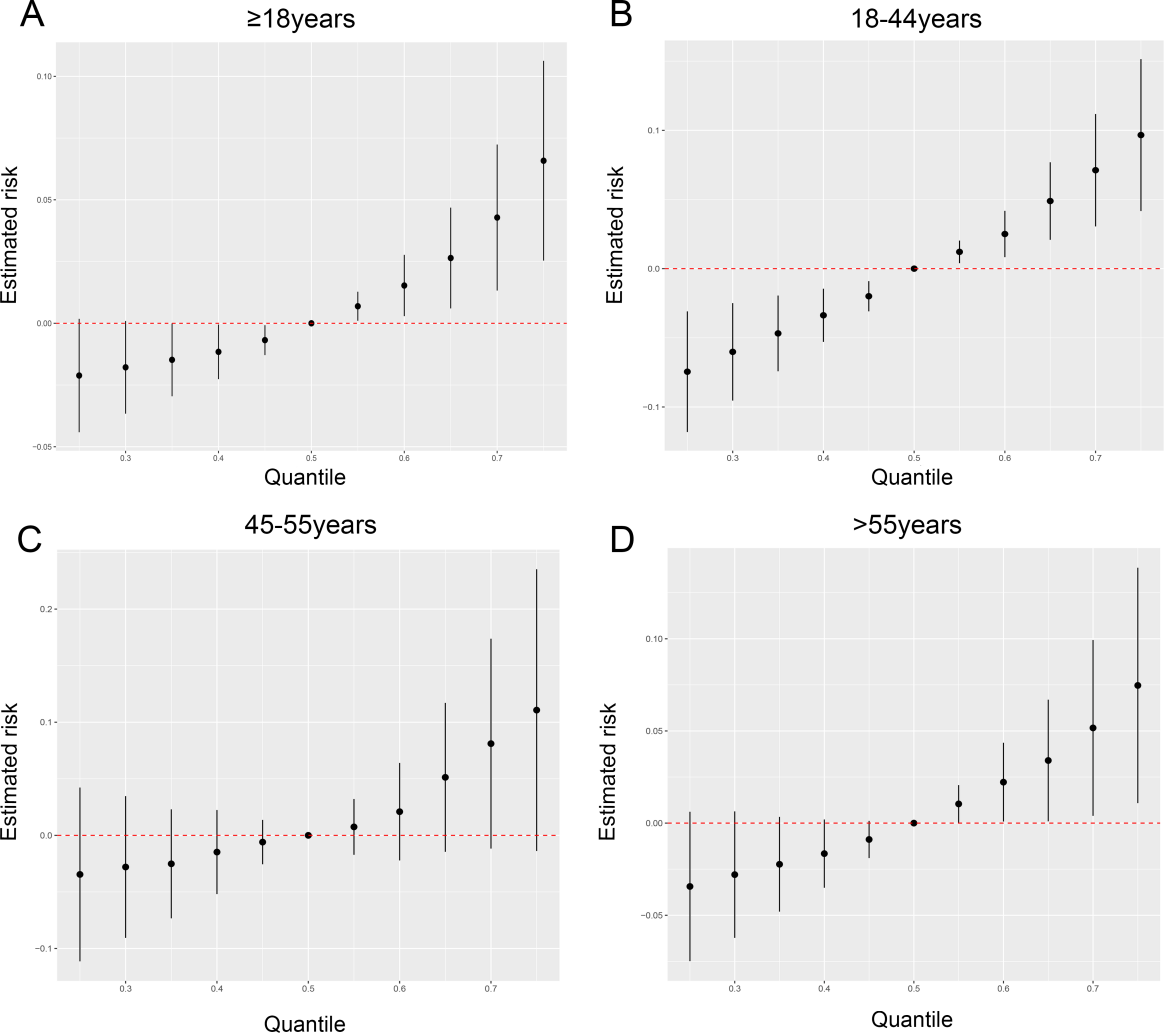
Association of metalloestrogen mixture exposure with depression in women. **(A)** Association of metalloestrogen mixture exposure with depression among whole women. **(B)** Association of metalloestrogen mixture exposure with depression among women aged 18-44 years. **(C)** Association of metalloestrogen mixture exposure with depression among women aged 45-55 years. **(D)** Association of metalloestrogen mixture exposure with depression among women aged >55 years.

What’s more, to further explore the role of metalloestrogens in depression, multiple metalloestrogens exposure model were be analyzed by using BKMR. When all seven metalloestrogens were set at a specific percentile (ranging from the 5th to the 95th percentile), the difference in depression levels was estimated in comparison to when these metalloestrogens were all at the 50th percentile. In **Figure 2A**, it is evident that among all women, the likelihood of depression rose when the concentrations of the metalloestrogens blend were at or above the 50th percentile. For women aged 18-44, there was a significant and positive correlation between metalloestrogens and depression risk (**Figure 2B**). Conversely, for women aged 45-55, metalloestrogens mixture did not show a significant association with the risk of depression (**Figure 2C**). As shown in **Figure 2D**, it is highlighted that the risk of depression in older age groups notably increased when the concentrations of metal mixtures were at or above the 60th percentile.

### 3.4 Oral contraceptive may influence the association of metalloestrogen mixture exposure with depression in women aged 18-44 years

**Figure 3:**
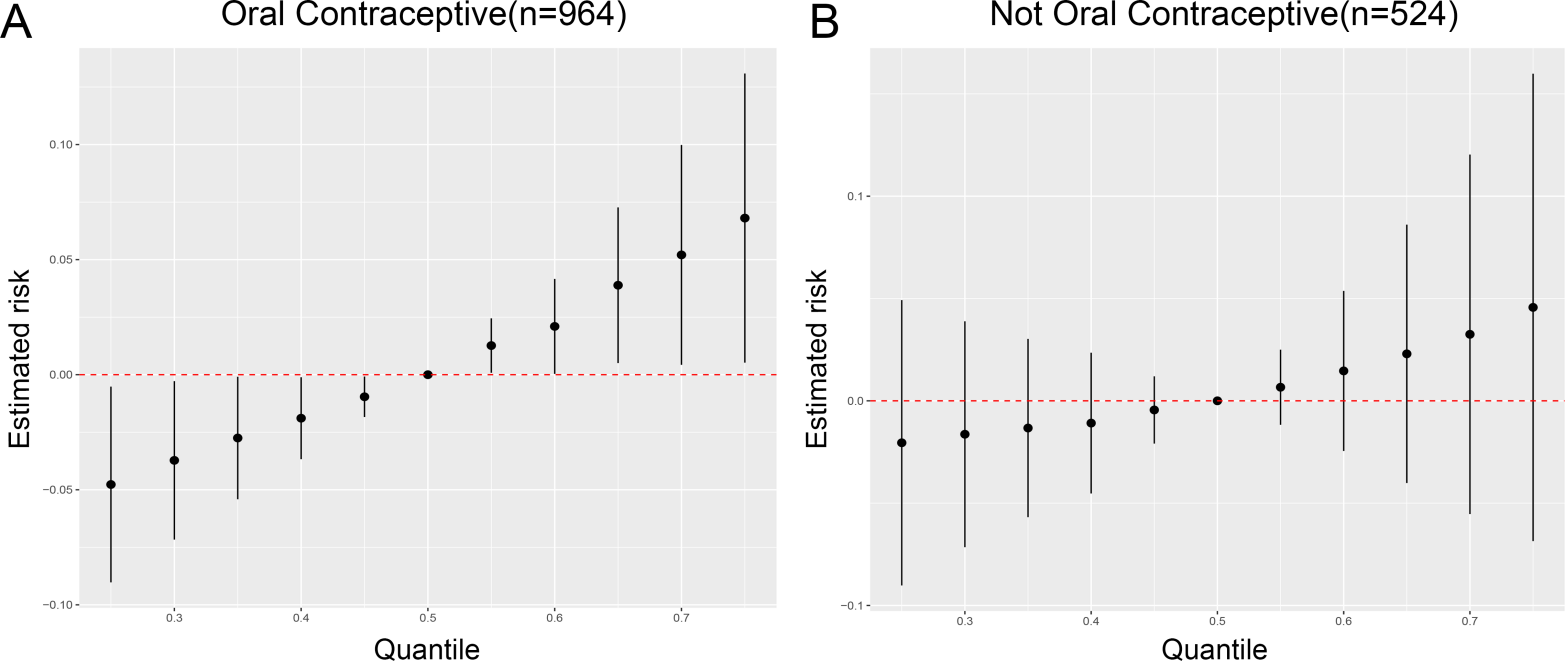
Oral contraceptive may influence the association of metalloestrogen mixture exposure with depression in women aged 18-44 years. **(A)** Association between metalloestrogens mixture exposure and depression during the women with a history of oral contraceptive use. **(B)** Association between metalloestrogens mixture exposure and depression during the women without a history of oral contraceptive use.

No significant association was found between oral contraceptive use and depression among women aged 18-44 years (**Supplementary Table S8**). However, BKMR analysis, when stratified by history of oral contraceptive use among women aged 18-44 years, revealed a significant positive association between metalloestrogens and depression in women with a history of oral contraceptive use (**Figure 3A**). Conversely, no such association was observed in women without a history of oral contraceptive use (**Figure 3B**).

## 4 Discussion

In this study, we firstly explored the risk of metalloestrogens exposure for depression in women across reproductive lifespan. Our results showed that exposure to Ba, Ca, Pb, Sb, and Sn alone was positively linked to depression risk in women. Conversely, Hg exposure was negatively correlated with depression risk in older women. Furthermore, BKMR analyses indicated that the depression risk in women of reproductive age could be influenced by exposure to a blend of metalloestrogens. The risk of depression significantly rose when the metalloestrogen mixture concentration reached or exceeded the 60th percentile in older women. Additionally, we observed that oral contraceptives might mediate the impact of metalloestrogens mixture exposure on depression in women during the reproductive stage.

Ba primarily enters the human body through water, food, and air, with industrial production, wastewater discharge, and fertilizer use also contributing to exposure (31). Our research discovered a positive association between Ba exposure and depression in women, particularly in perimenopausal women. In contrast, a previous study on depression in pregnant women did not find a link between Ba and maternal depression (32). This could be attributed to the unique nature of pregnancy, a special phase in a woman’s life characterized by significant hormonal and lifestyle changes (33). Perimenopausal women, however, undergo a gradual decline in physiological function, including irregular menstruation or its cessation. While hormone levels fluctuate, there is a gradual reduction in estrogen production by the ovaries (24). Further research is necessary to investigate the interplay between women’s physiological changes mediating Ba exposure and depression.

Cd is widely present in the environment as both an agricultural and industrial pollutant (34). The primary sources of Cd exposure for the population are smoking and diet (35). Cd can penetrate the blood-brain barrier, affecting glial cells and altering several molecular pathways, such as increasing lipid peroxidation, impairing antioxidant defenses, and lowering glutathione levels (21). Moreover, Cd can influence the monoaminergic neurotransmission system, which regulates mood states and plays a role in depression development (36, 37). Our study revealed a significant positive correlation between urinary Cd levels and depression risk. While consistent with previous research (19, 38), a study from China found no link between blood cadmium levels and depression incidence, particularly among the elderly (39). This discrepancy arises from differences in exposure characteristics. Blood Cd reflects recent and cumulative exposure, while urinary Cd indicates lifetime exposure, making urinary measurements the gold standard for assessing Cd exposure (40). Furthermore, demographic differences play a role. Our study focused on U.S. females at various reproductive stages, showing a stronger association between Cd exposure and depression in women during perimenopause and old age. Despite discrepancies, it is crucial to highlight the impact of Cd exposure on depression and implement effective intervention strategies to reduce its incidence.

For Mn, an essential micronutrient for life processes, plays a crucial role in various kinases and enzymes like glutathione lyase, guanidine aminopeptidase, glutamine synthetase, and superoxide dismutase (SOD) (41). For instance, Mn is a vital component of MnSOD, an antioxidant mitochondrial metalloenzyme that shields cells from oxidative stress (42). A prior cross-sectional study revealed that higher blood manganese levels in women were linked to a lower risk of depression (43). Furthermore, a recent meta-analysis indicated a negative correlation between dietary manganese intake and depression risk, suggesting that manganese supplementation could be a potential intervention to prevent depression (44). Our study, however, contrasts with these findings, possibly due to our use of urine samples and stricter inclusion criteria for females. Further research is required to investigate the connection between urinary Mn levels and depressive symptoms.

Pb, a highly toxic heavy metal, accumulates in bones over time and is released slowly (45). It disrupts enzyme activity and structural proteins, resulting in various harmful effects such as anemia, hypertension, cognitive deficits, immune imbalances, delayed bone and tooth development, as well as neurological and behavioral impacts (46). Our research indicates a strong link between Pb exposure and depression, and this association continues throughout a woman’s adult life. Recent studies have shown a positive association between blood Pb levels and depression in females aged 20–59 years (19). Furthermore, NHANES data from 2011–2012 revealed a connection between Pb exposure and depressive symptoms (47). However, a Korean study found no significant correlation between serum Pb levels and depression (38), possibly due to demographic and sample differences.

Sb is a crucial industrial raw material used in various valuable products such as flame retardants, paints, pigments, and electronics (48). Some studies consider it a novel neurotoxicant (49). We discovered a significant link between Sb exposure and depression risk, particularly among perimenopausal women. Research based on NHANES 2007–2016 also revealed a positive connection between urinary Sb levels and depressive symptoms in women (50). Sb induces autophagy through reactive oxygen species (ROS)-mediated cytotoxicity, with excessive autophagy potentially leading to neuronal apoptosis and depression (51). Animal studies demonstrated that prolonged Sb exposure increased levels of inflammatory factors (interleukin-1β (IL-1β), IL-6, and TNF-α) and pro-oxidant substances (glutathione peroxidase, malondialdehyde) (49), indicating that Sb boosts inflammatory responses, closely tied to depression (52).

Sn exists in both inorganic and organic forms, with the former used primarily as tin-plated cans and containers and in personal care products, such as stannous chloride, and the latter as a stabilizer and biocide for polyvinyl chloride, such as tributyltin (TBT) (53). Stannous chloride has been found in animal experiments to accelerate the release of transmitters from nerve endings in mice to promote neuromuscular transmission and thus stimulate or inhibit the central nervous system (54). TBT may alter levels of neurotransmitters, including dopamine and γ-aminobutyric acid, and affect gene expression related to mood, potentially contributing to instability and depression (55, 56). Additionally, TBT exposure can provoke inflammatory responses and increase oxidative stress, decreasing antioxidant levels, as seen in rat models (57). Our study aligns with previous findings (58), suggesting that high Sn levels are strongly associated with depression. Therefore, reducing Sn exposure levels may help prevent depression.

Hg, a common neurotoxin, is found in nature in elemental, organic, and inorganic forms (59). Human exposure to Hg primarily occurs through consuming fish, air pollution, and occupational settings (60). Hg can induce psychiatric symptoms by causing oxidative stress on the central nervous system, potentially disrupting serotonin metabolism (61). A study in South Korea revealed that elevated blood Hg levels, particularly in conjunction with low fish consumption, were linked to a higher risk of depression in Korean women (62). Interestingly, our study showed a contrasting result, indicating a negative correlation between urinary Hg levels and depression risk in older women. The consumption of fish and seafood, rich in polyunsaturated fatty acids (PUFA), could introduce variables affecting the link between urinary Hg and depression (63). Previous research has suggested that PUFA may have a role in preventing or treating depression (64). Moreover, older individuals, who often have a diet high in fish and nutrients, represent a unique demographic. Notably, the average urinary Hg concentration in our study was only 0.498 μg/L, significantly below the Environmental Protection Agency’s reference dose (65). Moving forward, further investigations are warranted to explore the relationship between consuming fish and seafood products and urinary Hg levels in the context of depression.

**Figure 4:**
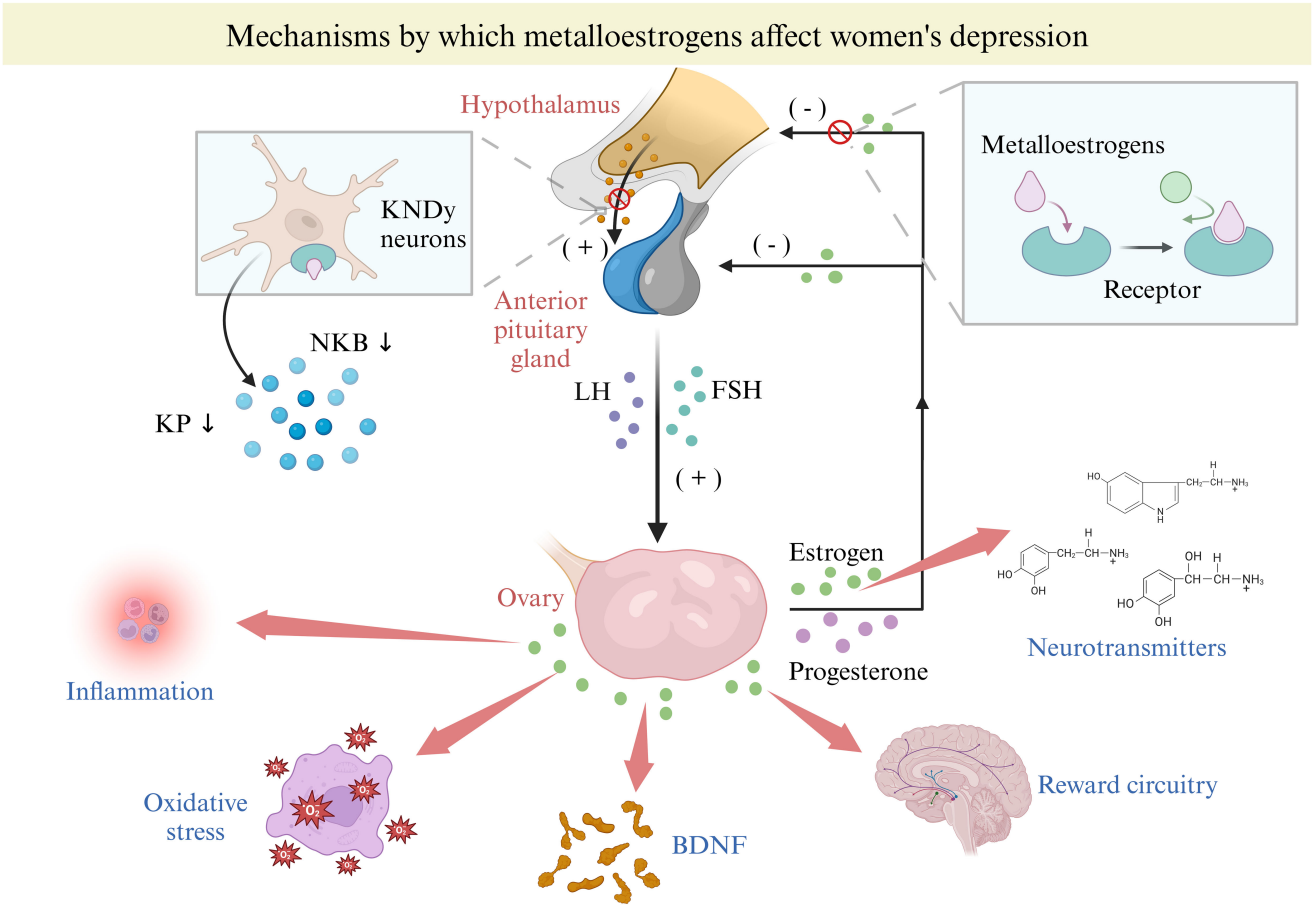
Mechanisms by which metalloestrogens affect women depression. BDNF, brain-derived neurotrophic factor; FSH, follicle-stimulating hormone; KDNy, Kisspeptin neurokinin B-dynorphin; KP, Kisspeptin; NKB, neuropeptides, neurokinin B.

Our previous research has shown that metalloestrogens have distinct mechanisms that affect the onset of depression (17), mainly through neuropeptide and epigenetic pathways (**Figure 4**). Kisspeptin (KP), a neuropeptide produced from the breakdown of a 145-amino-acid polypeptide precursor encoded by the KISS1/Kiss1 gene, plays a crucial role in regulating the hypothalamic-pituitary-gonadal axis (HPG) activity (66). Among the KP neurons situated in the arcuate nucleus of the hypothalamus is the Kisspeptin neurokinin B-dynorphin (KNDy) neuron, which not only produces KP but also co-produces two other neuropeptides, neurokinin B (NKB) and dynorphin (Dyn) (67, 68). Due to estrogen’s negative feedback on KNDy neurons, the activation of metalloestrogens with estrogen receptors on KNDy can suppress KP and NKB secretion while promoting dynorphin release (69). On the other hand, gonadotropin-releasing hormone (GnRH) neurons in the preoptic area are primarily influenced by circulating KP stimulation, leading to the release of endogenous GnRH (69). With inhibited KP secretion, reduced GnRH acts on the anterior pituitary, resulting in decreased gonadotropins (luteinizing hormone (LH) and follicle-stimulating hormone (FSH)) release (70). LH and FSH released further stimulate the ovaries to secrete E2 and progesterone, providing additional negative feedback on the KNDY neurons in the hypothalamus and the anterior pituitary (66, 71). Metalloestrogens disrupt the HPG axis by targeting KNDY neurons in the hypothalamus, leading to decreased E2 and progesterone secretion. This imbalance in E2 levels can heighten depression risk by impacting neurotransmitter production, inflammation, oxidative stress, reward circuits, and brain-derived neurotrophic factor (BDNF) (8–10).

Metalloestrogens can impact the expression of the KISS 1 gene through epigenetic mechanisms. Firstly, they can modify gene transcription through DNA methylation (72). Methylation in the promoter region or the first exon of the gene can silence gene activity (73), while demethylation can lead to gene reactivation. The promoter region of the KISS 1 gene is often methylated, potentially reducing KP expression (74). Secondly, acetylation, a common histone modification, along with metalloestrogens, can induce promoter deacetylation in KNDy neurons, contributing to the negative feedback of estrogen on KNDy (75). Lastly, microRNAs (miRNAs) are small non-coding RNAs approximately 22 bp in length that regulate gene expression post-transcriptionally by targeting specific sequences in mRNAs (76). They are involved in various health conditions, like inflammation and metabolic disorders (77). E2 can regulate miRNA transcription through estrogen receptors α and β (ERα and ERβ) in a tissue-specific and cell-dependent manner (78). A recent study showed that E2 can modify miRNAs post-injury, impacting genes linked to depression (79).

When stratified by women’s reproductive stages, it was observed that those in the reproductive period (18-44 years) showed higher vulnerability to metalloestrogens. The mixture of metalloestrogens was notably linked to an increased risk of depression in this group. Conversely, no significant correlation was detected in the perimenopausal phase (45-55 years old). This difference might stem from the normal estrogen levels within the reproductive stage, allowing the female brain to adjust to fluctuating estradiol levels and receptor activity (80). The impact of metalloestrogen exposure on the homeostatic balance appears to be more pronounced in women of reproductive age. In contrast, perimenopausal women undergo a gradual and irregular decline in estradiol levels, affecting the brain’s response to hormonal changes and potentially leading to an elevated risk of initial depressive episodes (81). Notably, external metalloestrogens seem to influence estrogen effects to a lesser extent than internal hormonal shifts in early perimenopause. As perimenopause progresses, the brain gradually adapts to these abnormal hormonal fluctuations, potentially clarifying the observed alleviation of perimenopausal symptoms (80). In older women, we noted a notable rise in the risk of depression in late life when the metalloestrogen mixture concentration reached or exceeded the 60th percentile. Estrogen deficiency in older women is caused by ovarian failure (24), and increased levels of metalloestrogen exposure worsen this condition, leading to significantly lower estrogen levels compared to typical older women, consequently elevating the depression risk in women.

BKMR, when stratified by history of oral contraceptive use among reproductive-age women (18-44 years), revealed an intriguing finding. It showed that for women with a history of oral contraceptive use, metalloestrogen mixtures were significantly and positively linked to depression. However, for women without a history of oral contraceptive use, there was no such association between metalloestrogen exposure and depression. Oral contraceptives act as exogenous steroids, leading to a chronic suppression of ovarian estradiol and progesterone production by disrupting the release of hypothalamic and pituitary hormones (82). Research indicates that women on hormonal contraception exhibit endogenous sex steroid levels akin to those seen in the early follicular phase of naturally cycling women (82). The presence of metalloestrogens further disrupts the homeostasis of endogenous sex steroids, rendering women vulnerable and heightening the likelihood of experiencing depression.

This study has numerous strengths. Firstly, we utilized a broader NHANES dataset across 4 cycles, along with a more comprehensive statistical analysis than prior studies, enabling a deeper understanding of the link between metalloestrogen exposure and depression risk in women. Secondly, we identified the connection between metalloestrogen exposure and depression risk based on women’s various reproductive cycles. Lastly, we explored the impact of contraceptive use on the relationship between metalloestrogen exposure and depression risk. However, there are several limitations to note. Firstly, this is a cross-sectional study, and different metals have varying half-lives. Secondly, to increase the sample size, our study only focused on metalloestrogens found in urine, excluding those in blood. Hence, future research should investigate the effects of blood-borne metalloestrogens. Then again, because the types of oral contraceptives were not specified in the database, we were unable to specify the specific ingredients in them to explore more deeply the mechanism of oral contraceptives in influencing the relationship between exposure to metalloestrogens and depression in women. Lastly, due to sample constraints, we did not examine the association between metalloestrogen exposure and depression risk in adolescent and pregnant women.

In conclusion, our study shows a strong link between metalloestrogen exposure and increased depression risk in adult women in the US. More research is necessary to validate these results and explore the biological mechanisms involved. This will aid in confirming the connection and promoting women’s mental health.

## Data Availability

The original contributions presented in the study are included in the article/**Supplementary Material**. Further inquiries can be directed to the corresponding authors.
