# Supplementary material for: Association of metalloestrogens exposure with depression in women across reproductive lifespan
Source: Front Psychiatry. 2024 Dec 3;15:1486402. doi: 10.3389/fpsyt.2024.1486402 (PMC11649658; doi:10.3389/fpsyt.2024.1486402)
Supplement: Supplementary file 1 [file Supplementaryfile1.docx]

**Supplementary Material**


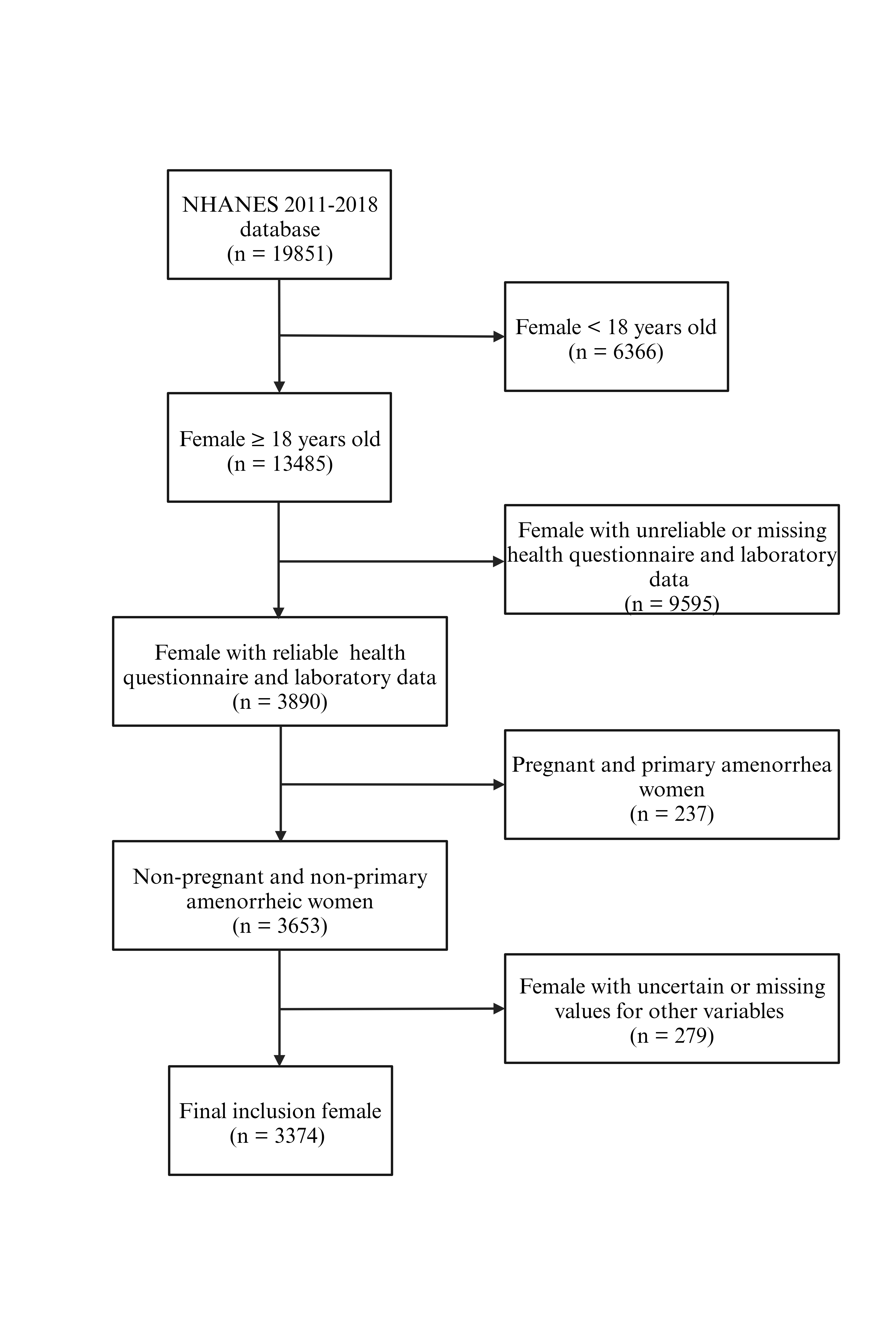


**Supplementary Figure 1.** Flowchart for selecting research participants.

**
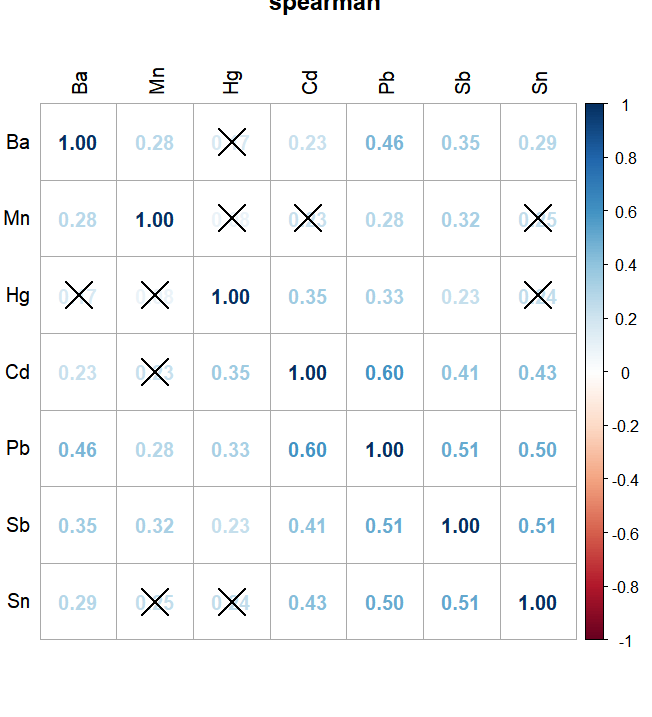
**

**Supplementary Figure 2.** Heatmap of the correlation of the seven metalloestrogens. The black color "х" indicates that there is no correlation between the two metalloestrogens.


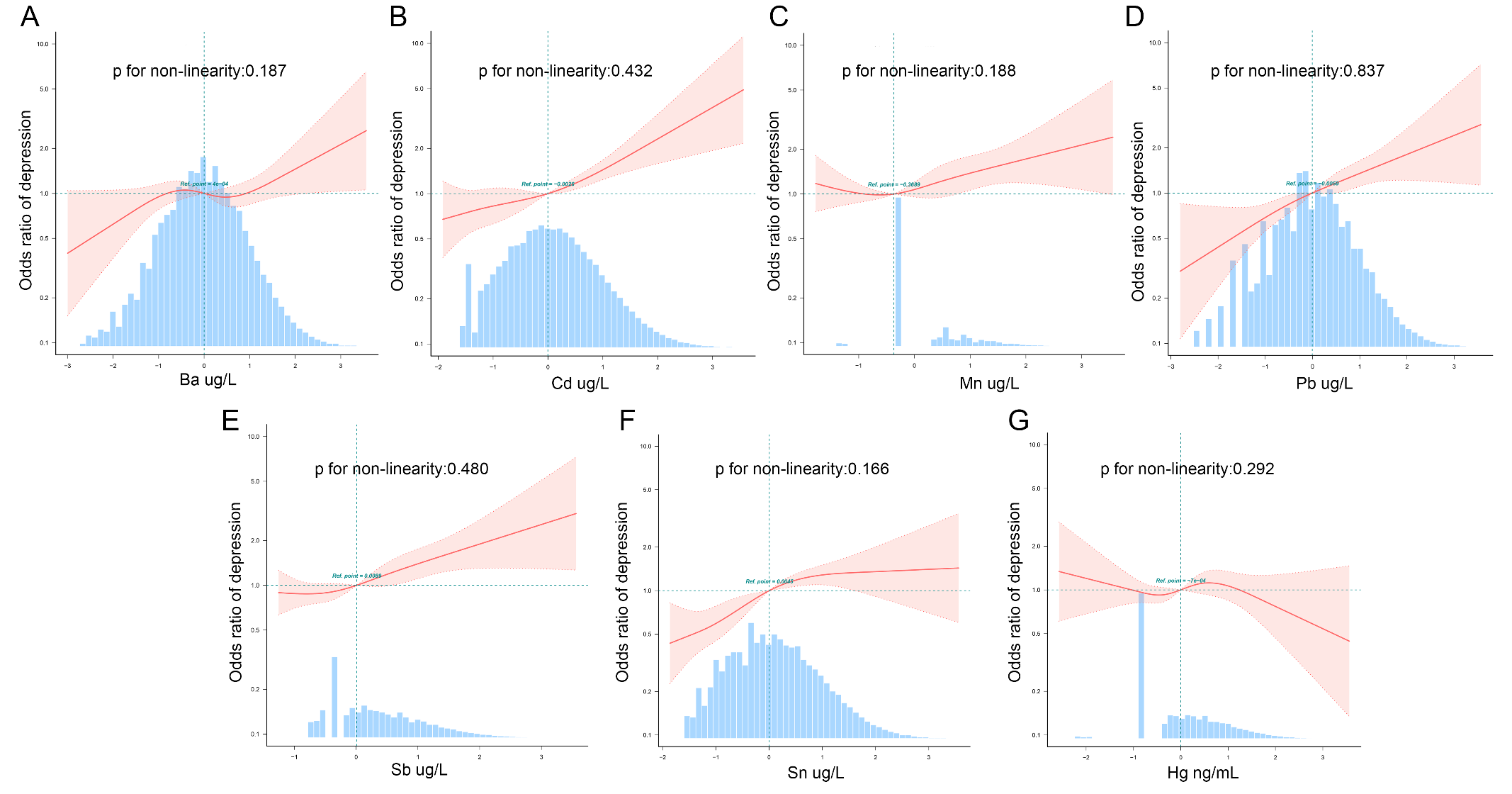


**Supplementary Figure 3.** Dose-response relationship between metalloestrogens and depression. 18-80 years old, all metalloestrogens converted to normal distribution using the normal score method.

**Supplementary Table 1.** Relative estrogenic efficiency of 7 metalloestrogens^1^

| Compounds | Relative efficiency (%) | Concentration |
| --- | --- | --- |
| 17 β-Estradiol | 100 | 1 nM |
| Sn | 93.5 | 100 nM |
| Cd | 73.8 | 1 μM |
| Sb | 60.9 | 1 μM |
| Ba | 46.8 | 1 μM |
| Pb | 25.0 | 1 μM |
| Mn | 9.1 | 1 μM |
| Hg | 4.4 | 1 μM |

Relative estrogenic efficiency is the percentage of compound and 17β-estradiol-induced luciferase activity calculated from estrogen receptor-dependent transcriptional expression analysis.

**Supplementary Table 2** After excluding the participants with hypertension histories, the association of metalloestrogens with depression, NHANES, 2011–2018.

| Variables | Crude OR (95%CI) | OR (95%CI)^1^ | OR (95%CI)^1^ | | | | P for trend |
| --- | --- | --- | --- | --- | --- | --- | --- |
| ug/L | Continuous | | Q1 | Q2 | Q3 | Q4 |  |
| Ba | 1.03 (0.98~1.08) | 1.02(0.97~1.07) | Reference | 1.17 (0.74~1.83) | 1.11 (0.70~1.75) | 1.07 (0.68~1.7) | 0.85 |
| Cd | **1.45 (1.09~1.91)*** | **1.51(1.12~2.04) ***** | Reference | 0.96 (0.59~1.56) | 1.23 (0.77~1.97) | **1.6 (1~2.54)*** | 0.026 |
| Mn | 1.00 (0.75~1.34) | 0.95(0.71~1.27) | Reference | 1.03 (0.6~1.79) | 1.17 (0.66~2.06) | **/** | 0.464 |
| Pb | **1.33 (1.12~1.57) ***** | **1.41(1.18~1.69) ***** | Reference | 1.36 (0.82~2.24) | **1.85 (1.15~2.97) **** | **1.91 (1.17~3.14) **** | 0.004 |
| Sb | **3.63 (1.25~10.59) **** | 3.22 (1.04~9.96) | Reference | 1.14 (0.71~1.83) | 1.08 (0.67~1.75) | **1.56 (1~2.45) **** | 0.059 |
| Sn | 1.01 (0.97~1.06) | 1.00 (0.95~1.05) | Reference | **1.84 (1.1~3.1) *** | **1.86 (1.1~3.14) *** | **2.71 (1.54~4.26) ***** | <0.001 |
| Hg ^2^ | 0.77 (0.57~1.03) | 0.78 (0.58~1.04) | Reference | 1.46 (0.44~4.82) | 1.37 (0.41~4.63) | 1.17 (0.34~3.98) | 0.408 |

Since the 25th percentile of Mn is equal to the 50th percentile, Mn is divided into three categorical variables.

^1^Adjusted for age, diabetes mellitus, household size, age at menarche, number of drinks in a year, and BMI. Q, quartile.

^2^ ng/ml.

**Supplementary Table 3.** After excluding the participants with hypertension histories, the association of metalloestrogens with depression after age subgroup, NHANES, 2011–2018.

| Variables | Crude OR (95%CI) | OR (95%CI)^1^ | OR (95%CI)^1^ | | | | P for trend |
| --- | --- | --- | --- | --- | --- | --- | --- |
| ug/L | Continuous | | Q1 | Q2 | Q3 | Q4 |  |
| Ba |  |  |  |  |  |  |  |
| 18-44 | 1.03 (0.98~1.09) | 1.03(0.98~1.09) | Reference | 0.85 (0.47~1.55) | 1.14 (0.65~2.01) | 1.02 (0.57~1.83) | 0.685 |
| 45-55 | 1.02 (0.86~1.21) | 1.01 (0.83~1.23) | Reference | 2.6 (0.81~8.32) | 1.6 (0.48~5.3) | 1.29 (0.37~4.49) | 0.97 |
| ≥56 | 1.03 (0.91~1.17) | 1.04 (0.91~1.18) | Reference | 1.27 (0.48~3.36) | 0.86 (0.31~2.4) | 1.02 (0.37~2.81) | 0.843 |
| Cd |  |  |  |  |  |  |  |
| 18-44 | 1.18 (0.59~2.35) | 1.21(0.58~2.51) | Reference | 1.05 (0.57~1.91) | 1.36 (0.76~2.42) | 1.25 (0.7~2.23) | 0.332 |
| 45-55 | **2.24 (1.33~3.78) **** | **2.4 (1.32~4.33) ***** | Reference | 0.97 (0.27~3.53) | 0.78 (0.21~2.84) | **2.82 (0.91~8.71) **** | 0.051 |
| ≥56 | 1.35 (0.87~2.08) | **1.31 (0.82~2.09)** | Reference | 2.62 (0.85~8.06) | 2.43 (0.76~7.82) | 2.06 (0.66~6.47) | 0.003 |
| Mn |  |  |  |  |  |  |  |
| 18-44 | 1.93 (0.84~4.46) | 1.86 (0.78~4.43) | Reference | 1.09 (0.55~2.18) | 1.2 (0.58~2.48) | / | 0.581 |
| 45-55 | 1 (0.65~1.53) | 1.02 (0.63~1.66) | Reference | 0.67 (0.2~2.32) | 1.31 (0.38~4.52) | / | 0.307 |
| ≥56 | 0.57 (0.08~4.12) | 0.87 (0.1~7.2) | Reference | 2.26 (0.46~10.96) | 1.9 (0.37~9.69) | / | 0.799 |
| Pb |  |  |  |  |  |  |  |
| 18-44 | **1.3 (1.04~1.63) **** | **1.42 (1.11~1.81) ***** | Reference | 2.09(1.03~4.22) | **2.89(1.45~5.73) ***** | **2.51(1.25~5.03) **** | 0.008 |
| 45-55 | 1.54 (0.91~2.6) | **1.56 (0.92~2.65) **** | Reference | 2.02 (0.49~8.33) | 3.51 (0.92~13.47) | 2.82 (0.72~11.15) | 0.107 |
| ≥56 | 1.28 (0.84~1.96) | 1.37 (0.87~2.16) | Reference | 1.26 (0.39~4.03) | 2.16 (0.75~6.2) | 2.13 (0.73~6.26) | 0.008 |
| Sb |  |  |  |  |  |  |  |
| 18-44 | 3.04 (0.77~12) | 2.75 (0.66~11.49) | Reference | 1.28 (0.68~2.41) | 1.15 (0.61~2.17) | **2.03 (1.13~3.62) **** | 0.021 |
| 45-55 | **19.34 (2~187.46) *** | **81.2 (4.43~1488.35) *** | Reference | 1.35 (0.4~4.49) | 1.15 (0.33~3.97) | 1.97 (0.63~6.21) | 0.276 |
| ≥56 | 0.67 (0~145.02) | 0.68 (0.01~56.16) | Reference | 0.56 (0.23~1.39) | 0.84 (0.37~1.91) | / | 0.672 |
| Sn |  |  |  |  |  |  |  |
| 18-44 | 1.02 (0.95~1.1) | 1.01 (0.95~1.09) | Reference | **3.58 (1.72~7.48) **** | **2.78 (1.32~5.86) **** | **3.29 (1.56~6.93) **** | 0.014 |
| 45-55 | 1.09 (0.99~1.21) | 1.11 (1~1.24) | Reference | 1.68 (0.39~7.21) | 1.75 (0.41~7.53) | **5.06 (1.31~19.54) **** | <0.001 |
| ≥56 | 0.93 (0.79~1.1) | 0.92 (0.77~1.1) | Reference | 2.11 (0.67~6.6) | 1.45 (0.43~4.95) | **3.05 (1.01~9.15) **** | <0.001 |
| Hg ^2^ |  |  |  |  |  |  |  |
| 18-44 | 0.77 (0.51~1.16) | 0.79 (0.52~1.18) | Reference | 1.81 (0.41~7.9) | 1.82 (0.41~8.11) | 1.41 (0.31~6.33) | 0.571 |
| 45-55 | 0.87 (0.55~1.37) | 0.87 (0.54~1.4) | Reference | 0.54 (0.2~1.45) | 1.04 (0.42~2.55) | / | 0.955 |
| ≥56 | 0.59 (0.26~1.33) | 0.57 (0.24~0.197) | Reference | 1.79 (0.75~4.25) | 0.86 (0.33~2.39) | / | 0.776 |

Since the 25th percentile of Mn,Hg,Sb is equal to the 50th percentile, Mn,Hg,Sb is divided into three categorical variables.

^1^Adjusted for diabetes mellitus, household size, age at menarche, number of drinks in a year, and BMI. Q, quartile.

^2^ ng/ml.

**Supplementary Table 4.**After excluding the participants with diabetes histories, the association of metalloestrogens with depression, NHANES, 2011–2018.

| Variables | Crude OR (95%CI) | OR (95%CI)^1^ | OR (95%CI)^1^ | | | | P for trend |
| --- | --- | --- | --- | --- | --- | --- | --- |
| ug/L | Continuous | | Q1 | Q2 | Q3 | Q4 |  |
| Ba | **1.07 (1.02~1.12) **** | 1.07 (1.02~1.12) | Reference | 1.25 (0.85~1.84) | 1.33 (0.91~1.95) | 1.33 (0.91~1.95) | 0.14 |
| Cd | **1.49 (1.19~1.86) ***** | **1.5 (1.18~1.91) ***** | Reference | 0.93 (0.62~1.4) | 1.32 (0.9~1.94) | **1.61 (1.09~2.37)**** | 0.004 |
| Mn | 1.06 (0.83~1.34) | 1.02(0.8~1.32) | Reference | 1.09 (0.67~1.76) | 1.31(0.8~2.14) | **/** | 0.149 |
| Pb | **1.4 (1.2~1.64) ***** | **1.48 (1.25~1.74) ***** | Reference | 1.33 (0.88~2) | **1.66 (1.11~2.47) *** | **2.05 (1.38~3.04) **** | <0.001 |
| Sb | **2.77 (1.22~6.32) *** | 2.06 (0.85~4.97) | Reference | 0.88 (0.59~1.32) | 1.02 (0.69~1.51) | **1.52 (1.05~2.18) *** | 0.01 |
| Sn | 1 (0.97~1.04) | 1 (0.96~1.03) | Reference | 1.35 (0.88~2.06) | **1.78 (1.19~2.67) **** | **2.04(1.36~3.05) ***** | <0.001 |
| Hg ^2^ | 0.9 (0.74~1.08) | 0.9 (0.75~1.08) | Reference | 0.84 (0.37~1.91) | 1 (0.44~2.31) | 0.8 (0.35~1.86) | 0.866 |

Since the 25th percentile of Mn is equal to the 50th percentile, Mn is divided into three categorical variables.

^1^Adjusted for age, hypertension, household size, age at menarche, number of drinks in a year, and BMI. Q, quartile.

^2^ ng/ml.

**Supplementary Table 5.** After excluding the participants with diabetes histories, the association of metalloestrogens with depression after age subgroup, NHANES, 2011–2018.

| Variables | Crude OR (95%CI) | OR (95%CI)^1^ | OR (95%CI)^1^ | | | | P for trend |
| --- | --- | --- | --- | --- | --- | --- | --- |
| ug/L | Continuous | | Q1 | Q2 | Q3 | Q4 |  |
| Ba |  |  |  |  |  |  |  |
| 18-44 | **1.06 (1~1.13) *** | 1.06 (0.99~1.13) | Reference | 1.29 (0.76~2.2) | 1.13 (0.65~1.96) | 1.09 (0.63~1.89) | 0.927 |
| 45-55 | 1.11(1~1.24) | 1.11 (0.99~1.25) | Reference | 1.83 (0.74~4.52) | 1.86 (0.79~4.4) | 1.75 (0.74~4.16) | 0.247 |
| ≥56 | 1.07 (0.97~1.17) | 1.07 (0.98~1.18) | Reference | 0.94 (0.45~1.99) | 1.29 (0.64~2.59) | 1.64 (0.82~3.27) | 0.102 |
| Cd |  |  |  |  |  |  |  |
| 18-44 | 1.49 (0.86~2.56) | 1.38 (0.75~2.54) | Reference | 0.97 (0.55~1.71) | 1.21 (0.69~2.1) | 1.46 (0.85~2.5) | 0.106 |
| 45-55 | **1.72 (1.11~2.67) *** | **1.69 (1.04~2.74) *** | Reference | 1.49 (0.58~3.79) | 1.8 (0.71~4.57) | 2.4 (0.99~5.83) | 0.043 |
| ≥56 | **1.47 (1.08~2.01) *** | **1.49 (1.08~2.06) *** | Reference | 0.68 (0.31~1.49) | 1.14 (0.57~2.29) | 1.61 (0.83~3.11) | 0.063 |
| Mn |  |  |  |  |  |  |  |
| 18-44 | 1.87 (0.86~4.06) | 1.75 (0.77~3.97) | Reference | 1.23 (0.62~2.44) | 1.48 (0.73~2.99) | / | 0.216 |
| 45-55 | 0.97 (0.62~1.51) | 1.01 (0.59~1.74) | Reference | 0.7(0.26~1.91) | 1.12 (0.41~3.11) | / | 0.352 |
| ≥56 | 1.28 (0.59~2.76) | 1.2 (0.55~2.64) | Reference | 1.54 (0.58~4.07) | 1.53 (0.56~4.17) | / | 0.573 |
| Pb |  |  |  |  |  |  |  |
| 18-44 | **1.33 (1.07~1.65) **** | **1.46 (1.15~1.85) ***** | Reference | **1.86 (1~3.46) *** | **2.43 (1.34~4.4) **** | **2.24(1.23~4.09) **** | 0.007 |
| 45-55 | 1.62(0.98-2.68) | 1.64 (0.98~2.73) | Reference | 1.41 (0.55~3.66) | 1.78 (0.71~4.45) | 2.39 (0.97~5.9) | 0.044 |
| ≥56 | **1.52 (1.13~2.04) **** | **1.53 (1.13~2.07) **** | Reference | 0.99 (0.46~2.14) | 1.68 (0.84~3.37) | 1.57 (0.77~3.19) | 0.101 |
| Sb |  |  |  |  |  |  |  |
| 18-44 | **4.03 (1.15~14.13)*** | 3.33 (0.92~12.09) | Reference | 1.24 (0.69~2.23) | 0.98 (0.53~1.82) | **2.24 (1.31~3.82) **** | 0.003 |
| 45-55 | **7.4 (1.09~50.5) *** | 7.18 (0.78~65.9) | Reference | 0.84(0.34~2.09) | 1.49 (0.65~3.43) | 1.31 (0.58~2.97) | 0.317 |
| ≥56 | 1.79 (0.16~20.03) | 1.43 (0.07~29.82) | Reference | 0.85 (0.41~1.76) | 0.99 (0.49~2) | 1.16 (0.59~2.27) | 0.563 |
| Sn |  |  |  |  |  |  |  |
| 18-44 | 1.01 (0.96~1.06) | 1 (0.95~1.06) | Reference | **2.73 (1.43~5.2) **** | **2.62 (1.37~4.98) **** | **2.57 (1.34~4.93) **** | 0.017 |
| 45-55 | 1.03 (0.98~1.08) | 1.01 (0.96~1.07) | Reference | 1.22 (0.46~3.27) | 1.65 (0.65~4.19) | **2.52 (1.03~6.17) *** | 0.023 |
| ≥56 | 0.98 (0.91~1.06) | 0.97 (0.9~1.05) | Reference | 0.85 (0.38~1.9) | **2.16 (1.07~4.38) *** | 1.52 (0.73~3.17) | 0.063 |
| Hg ^2^ |  |  |  |  |  |  |  |
| 18-44 | 0.88 (0.64~1.2) | 0.88 (0.65~1.21) | Reference | 1.27 (0.37~4.34) | 1.11(0.31~3.9) | 1.13 (0.32~3.97) | 0.674 |
| 45-55 | 0.93 (0.72~1.21) | 0.94 (0.75~1.19) | Reference | 0.82 (0.09~7.42) | 1 (0.11~9.29) | 0.83 (0.09~7.86) | 0.908 |
| ≥56 | 0.82 (0.54~1.25) | 0.85 (0.58~1.24)) | Reference | 1.31 (0.73~2.33) | 0.94 (0.5~1.74) | / | 0.852 |

Since the 25th percentile of Mn,Hg is equal to the 50th percentile, Mn,Hg is divided into three categorical variables.

^1^Adjusted for hypertension, household size, age at menarche, number of drinks in a year, and BMI. Q, quartile.

^2^ ng/ml.

**Supplementary Table 6.** After excluding the participants with diabetes and hypertension histories, the association of metalloestrogens with depression, NHANES, 2011–2018.

| Variables | Crude OR (95%CI) | OR (95%CI)^1^ | OR (95%CI)^1^ | | | | P for trend |
| --- | --- | --- | --- | --- | --- | --- | --- |
| ug/L | Continuous | | Q1 | Q2 | Q3 | Q4 |  |
| Ba | **1.06 (1~1.12) *** | 1.06(1~1.12) | Reference | 1.09 (0.66~1.78) | 1.21 (0.75~1.96) | 1.12 (0.68~1.83) | 0.584 |
| Cd | 1.35 (0.99~1.83) | **1.46(1.06~2.02) **** | Reference | 1.04(0.63~1.74) | 1.38 (0.85~2.26) | 1.55 (0.94~2.57) | <0.05 |
| Mn | 1.01 (0.73~1.41) | 0.97(0.69~1.36) | Reference | 1.17 (0.64~2.13) | 1.26 (0.67~2.34) | / | 0.481 |
| Pb | **1.34 (1.13~1.59) ***** | **1.42(1.18~1.71) ***** | Reference | 1.2 (0.71~2.03) | **1.68 (1.01~2.8) *** | **1.74 (1.03~2.94) **** | <0.05 |
| Sb | **3.4(1.13~10.27) **** | 2.86 (0.88~9.26) | Reference | 1.1 (0.66~1.84) | 1.08 (0.65~1.81) | 1.5 (0.93~2.42) | 0.099 |
| Sn | 1.02 (0.97~1.07) | 1.02 (0.96~1.07) | Reference | **1.92 (1.11~3.33) *** | **1.84 (1.06~3.19) *** | **2.41 (1.41~4.12) ***** | <0.01 |
| Hg ^2^ | 0.76 (0.55~1.05) | 0.76 (0.55~1.06) | Reference | 1.46 (0.44~4.85) | 1.37 (0.4~4.64) | 1.16 (0.34~3.98) | 0.431 |

Since the 25th percentile of Mn is equal to the 50th percentile, Mn is divided into three categorical variables.

^1^Adjusted for age, household size, age at menarche, number of drinks in a year, and BMI. Q, quartile.

^2^ ng/ml.

**Supplementary Table 7.** After excluding the participants with diabetes and hypertension histories, the association of metalloestrogens with depression after age subgroup, NHANES, 2011–2018.

| Variables | Crude OR (95%CI) | OR (95%CI)^1^ | OR (95%CI)^1^ | | | | P for trend |
| --- | --- | --- | --- | --- | --- | --- | --- |
| ug/L | Continuous | | Q1 | Q2 | Q3 | Q4 |  |
| Ba |  |  |  |  |  |  |  |
| 18-44 | 1.06 (0.98~1.14) | 1.07(0.99~1.15) | Reference | 0.92(0.51~1.68) | 1.13(0.63~2) | 0.94 (0.52~1.71) | 0.976 |
| 45-55 | 1.07 (0.92~1.25) | 1.08 (0.91~1.28) | Reference | 2.61(0.63~10.8) | 2.53 (0.63~10.11) | 2.25 (0.54~9.32) | 0.264 |
| ≥56 | 1.06 (0.94~1.2) | 1.05 (0.93~1.1) | Reference | 0.99 (0.31~3.17) | 0.83 (0.24~2.79) | 1.35 (0.45~4.03) | 0.088 |
| Cd |  |  |  |  |  |  |  |
| 18-44 | 1.2 (0.59~2.41) | 1.22(0.58~2.56) | Reference | 1.01(0.55~1.86) | 1.21(0.67~2.19) | 1.22(0.68~2.2) | 0.409 |
| 45-55 | **2.1 (1.21~3.63) **** | **2.04(1.11~3.75) *** | Reference | 1.21 (0.3~4.86) | 1.03 (0.26~4.11) | 2.92(0.85~10.04) | 0.082 |
| ≥56 | 1.28 (0.77~2.13) | 1.29(0.73~2.28) | Reference | 1.86 (0.52~6.67) | 1.43 (0.38~5.43) | 1.57 (0.43~5.72) | 0.665 |
| Mn |  |  |  |  |  |  |  |
| 18-44 | 1.99 (0.87~4.58) | 1.088(0.79~4.47) | Reference | 1.19(0.58~2.44) | 1.26(0.59~2.68) | / | 0.331 |
| 45-55 | 0.98 (0.59~1.62) | 1.01 (0.56~1.8) | Reference | 0.94 (0.23~3.8) | 1.56 (0.38~6.51) | / | 0.347 |
| ≥56 | 0.03 (0~15.37) | 0.02 (0~13.68) | Reference | 2.96 (0.35~24.85) | 2.83 (0.25~19.9) | / | 0.9 |
| Pb |  |  |  |  |  |  |  |
| 18-44 | **1.31 (1.04~1.64) *** | **1.43(1.12~1.83) **** | Reference | **2.04(1.01~4.13)*** | **2.58(1.29~5.18) **** | **2.41(1.2~4.85) **** | 0.015 |
| 45-55 | 1.68(0.88~3.22) | 1.56(0.87~2.78) | Reference | 2.89 (0.55~15.19) | 3.38 (0.66~17.22) | 4.2 (0.84~20.91) | 0.084 |
| ≥56 | 1.35 (0.87~2.11) | 1.35 (0.83~2.19) | Reference | 1.61 (0.44~5.86) | 1.39 (0.41~4.75) | 1.5(0.44~5.13) | 0.597 |
| Sb |  |  |  |  |  |  |  |
| 18-44 | 3.12 (0.79~12.33) | 2.76 (0.66~11.49) | Reference | 1.26(0.66~2.39) | 1.16 (0.6~2.22) | **2.02(1.11~3.65) *** | 0.024 |
| 45-55 | **13.83(1.24~154) *** | **49.61(2.29~1073) *** | Reference | 1.39 (0.36~5.35) | 1.24 (0.32~4.82) | 2.11 (0.6~7.43) | 0.259 |
| ≥56 | 0.06 (0~1445) | 0.01 (0~505.91) | Reference | 0.4 (0.13~1.22) | 0.51 (0.19~1.39) | / | 0.188 |
| Sn |  |  |  |  |  |  |  |
| 18-44 | 1.04 (0.96~1.13) | 1.04 (0.96~1.14) | Reference | **3.31(1.57~6.96) **** | **2.73(1.29~5.77) **** | **3.15(1.51~6.7) **** | 0.014 |
| 45-55 | 1.09(0.98~1.2) | 1.11 (0.99~1.24) | Reference | 1.56 (0.35~7.01) | 1.47 (0.33~6.58) | **4.22(1.07~16.68) *** | 0.026 |
| ≥56 | 0.91 (0.69~1.18) | 0.91 (0.69~1.18) | Reference | 1.45 (0.38~5.48) | 0.98 (0.23~4.21) | 2.55 (0.74~8.81) | 0.165 |
| Hg ^2^ |  |  |  |  |  |  |  |
| 18-44 | 0.75 (0.48~1.17) | 0.76(0.49~1.2) | Reference | 1.81 (0.41~7.93) | 1.74(0.39~7.77) | 1.38(0.31~6.23) | 0.516 |
| 45-55 | 0.94 (0.63~1.39) | 0.92 (0.58~1.47) | Reference | 0.67 (0.07~6.46) | 0.61 (0.06~6.24) | 0.55 (0.05~5.79) | 0.62 |
| ≥56 | 0.41 (0.12~1.38) | 0.35 (0.09~1.26) | Reference | 1.72 (0.63~4.67) | 0.62 (0.19~2.08) | / | 0.354 |

Since the 25th percentile of Mn,Hg,Sb is equal to the 50th percentile, Mn,Hg,Sb is divided into three categorical variables.

^1^Adjusted for household size, age at menarche, number of drinks in a year, and BMI. Q, quartile.

^2^ ng/ml.

**Supplementary Table 8.** Association between oral contraceptives and depression among women aged 18-44 years.

| Variables |  | Crude OR (95%CI) | OR (95%CI)^1^ |
| --- | --- | --- | --- |
| Oral contraceptive | No | Reference | Reference |
|  | Yes | 1.46 (0.99~2.16) | 1.31 (0.87~1.98) |

^1^Adjusted for age, diabetes mellitus, hypertension, household size, age at menarche, number of drinks in a year, and BMI.

References:

1. Choe SY, Kim SJ, Kim HG, et al. Evaluation of estrogenicity of major heavy metals. *Sci Total Environ* 2003;312(1-3):15-21. doi: 10.1016/S0048-9697(03)00190-6 [published Online First: 2003/07/23]
